# *In vitro* effects of commercial mouthwashes on several virulence traits of *Candida albicans*, viridans streptococci and *Enterococcus faecalis* colonizing the oral cavity

**DOI:** 10.1371/journal.pone.0207262

**Published:** 2018-11-15

**Authors:** Andrea Ardizzoni, Eva Pericolini, Simona Paulone, Carlotta Francesca Orsi, Anna Castagnoli, Ilaria Oliva, Elena Strozzi, Elisabetta Blasi

**Affiliations:** 1 Department of Surgical, Medical, Dental and Morphological Sciences with Interest in Transplant, Oncological and Regenerative Medicine, University of Modena and Reggio Emilia, Modena, Italy; 2 Graduate School of Microbiology and Virology, University of Modena and Reggio Emilia, Modena, Italy; Louisiana State University, UNITED STATES

## Abstract

Oral microbiota consists of hundreds of different species of bacteria, fungi, protozoa and archaea, important for oral health. Oral mycoses, mostly affecting mucosae, are mainly caused by the opportunistic pathogen *Candida albicans*. They become relevant in denture-wearers elderly people, in diabetic patients, and in immunocompromised individuals. Differently, bacteria are responsible for other pathologies, such as dental caries, gingivitis and periodontitis, which affect even immune-competent individuals. An appropriate oral hygiene can avoid (or at least ameliorate) such pathologies: the regular and correct use of toothbrush, toothpaste and mouthwash helps prevent oral infections. Interestingly, little or no information is available on the effects (if any) of mouthwashes on the composition of oral microbiota in healthy individuals. Therefore, by means of *in vitro* models, we assessed the effects of alcohol-free commercial mouthwashes, with different composition (4 with chlorhexidine digluconate, 1 with fluoride, 1 with essential oils, 1 with cetylpyridinium chloride and 1 with triclosan), on several virulence traits of *C*. *albicans*, and a group of viridans streptococci, commonly colonizing the oral cavity. For the study here described, a reference strain of *C*. *albicans* and of streptococci isolates from pharyngeal swabs were used. Chlorhexidine digluconate- and cetylpyridinium chloride-containing mouthwashes were the most effective in impairing *C*. *albicans* capacity to adhere to both abiotic and biotic surfaces, to elicit proinflammatory cytokine secretion by oral epithelial cells and to escape intracellular killing by phagocytes. In addition, these same mouthwashes were effective in impairing biofilm formation by a group of viridans streptococci that, notoriously, cooperate with the cariogenic *S*. *mutans*, facilitating the establishment of biofilm by the latter. Differently, these mouthwashes were ineffective against other viridans streptococci that are natural competitors of *S*. *mutans*. Finally, by an *in vitro* model of mixed biofilm, we showed that mouthwashes-treated *S*. *salivarius* overall failed to impair *C*. *albicans* capacity to form a biofilm. In conclusion, the results described here suggest that chlorhexidine- and cetylpyridinium-containing mouthwashes may be effective in regulating microbial homeostasis of the oral cavity, by providing a positive balance for oral health. On the other side, chlorhexidine has several side effects that must be considered when prescribing mouthwashes containing this molecule.

## Introduction

*Candida albicans* is a commensal microorganism of humans: it dwells in the gastro-intestinal tract, oral and vaginal mucosa of most healthy individuals. Sometimes, it behaves as an opportunistic pathogen, thus causing symptomatic mucosal infections.

*C*. *albicans* is characterized by several developmental cell types, including yeast and filamentous forms (pseudohyphae and hyphae). Filaments are distinct from yeast-form cells in cell wall structure, cell wall proteins, transcriptional programs and recognition/interaction with immune system [1,2]. The ability of this species to switch between the yeast and the filamentous forms is strongly associated with virulence. By *ex vivo* models of candidiasis [3–5], the hyphal form of the fungus has been shown to cause more tissue damage than the yeast-form, because it grants fungal ability to adhere to epithelial surfaces, form biofilm, elicit proinflammatory cytokines production and avoid phagocytosis and/or intracellular killing.

Among several mechanisms, cell surface hydrophobicity plays an important role in the adhesion of *C*. *albicans* to inert surfaces. This adhesion capacity is one of the main predisposing factors to oral infections, because abiotic materials such as acrylic denture base [6,7], orthodontic metal braces [8] and surfaces of dental restorations [9] are often present inside the oral cavity. Therefore, the ability of *C*. *albicans* to adhere to inert materials of this kind would explain why *Candida* stomatitis and other fungal oral infections affect about 67% of elderly denture wearers [6,7,9,10]. Not only is *C*. *albicans* able to bind to inert materials, but it can also bind to biotic surfaces in the oral cavity (mucosal epithelia and teeth surface [11]) by means of additional mechanisms, such as interactions between epithelial receptors and Candida adhesins [12].

Epithelial cells produce a variety of cytokines in response to Candida infection, including Granulocyte-Colony Stimulating Factor (G-CSF), Granulocyte Macrophage-Colony Stimulating Factor (GM-CSF), Interleukin-1α (IL-1α), Interleukin-1β (IL-1β) and Interleukin-6 (IL-6), as well as the chemokines Regulated on Activation, Normal T Cell Expressed and Secreted (RANTES), Interleukin-8 (IL-8) and Macrophage Inflammatory Protein 3α (MIP3α) [13,14].

Oral-pharyngeal candidiasis, mainly associated with *C*. *albicans* species, is common amongst AIDS patients, where it is considered a marker of disease development in HIV positive individuals. Furthermore, oral-pharyngeal candidiasis is often associated with oral cancer, it can develop in individuals that use dental prostheses (especially elderly people), and it frequently affects diabetic patients, as well as terminally ill patients who fail to produce sufficient saliva [15]. In several cases, oral candidiasis may be prevented by a good oral hygiene, including the daily use of toothbrush and mouthwashes (MoWs). By means of an *in vitro* model, we have recently demonstrated that both *C*. *albicans* hyphal development and biofilm formation/persistence are affected by MoWs, provided that they contain chlorhexidine digluconate (CHX) [16].

In addition to fungi, more than 700 species of bacteria have been identified and/or predicted to habit within the oral cavity [17]. Actually, bacteria are the main component of the oral microbiota. Among them, many species belonging to the genus *Streptococcus* have been described and their taxonomic relations have been unravelled by 16S rRNA gene sequence comparisons. By such method, streptococci have been divided in 6 different groups: pyogenic, mitis, anginosus, mutans, salivarius, and bovis [18]. With the exception of the *Streptococcus mutans* species (the main etiological agent of dental caries), oral streptococci are mainly considered avirulent or even beneficial organisms. In some cases, certain oral streptococci can even hinder the development of a cariogenic *S*. *mutans* biofilm [19]. Recently, attention has been focused on several components of the mitis group (*S*. *gordonii*, *S*. *oralis*, *S*. *mitis*, *S*. *parasanguinis*, *S*. *sanguinis*), which have been implicated in supporting a pathogenic multispecies biofilm, given their ability to co-aggregate with other bacterial species and fungi as well. Not only such co-aggregation has been shown to confer a mutual advantage in biofilm formation, but also interactions among these microorganisms have been hypothesized to play a role during both commensal colonization of oral surfaces and in the onset and development of opportunistic infections [20].

*E*. *faecalis*, which is another microbial component of the oral microbiota, is also important for oral health, since it is one of the species mainly involved in failures of endodontic treatments [21,22]. *E*. *faecalis* localizes in dentinal canaliculi of devitalized teeth, resists to common disinfectants and endodontic irrigants [23,24], and can survive for long periods even under starving conditions, until changes of root canal microenvironment will allow its re-growth [25,26]. For bacteria, as well as for *Candida*, the biofilm production is an important virulence trait, especially in an ecological setting like the oral cavity, where many microbial species share the same ecological niche, compete for spaces and nutrients and therefore influence each other. In particular, reciprocal interaction among different species of streptococci can be of importance for the onset of oral diseases, such as carious lesions.

Not only some non-*mutans* streptococci, but also fungi may play a relevant role in the onset of carious lesions. *C*. *albicans*, in particular, has been hypothesized to significantly contribute to caries pathogenesis, especially in children, adolescents and young adults, because of its acidogenicity, capability to form hyphae and to secrete dentine-degrading enzymes [27,28]. In addition, both *in vitro* [29] and *in vivo* [30,31] experimental evidence suggests that, within the oral cavity, *Candida*-Streptococcal interactions do occur. Such interactions result in the formation of multi-species biofilm communities, where microorganisms use *quorum sensing* mechanisms to communicate with each other, to adjust their population density and to modulate gene expression patterns, in order to better adapt to the microenvironment where they dwell [20].

Following the observations recently described [16], this study was aimed to expand our knowledge about MoWs effects on several components of the oral microbiota. In particular, by means of *in vitro* models, we investigated the behaviour of MoWs-treated *C*. *albicans* in terms of: a) adhesion to both abiotic and biotic surfaces, b) capacity to elicit pro-inflammatory response by epithelial cells and c) susceptibility to phagocytosis and intracellular killing, as assessed by phagolysosome acidification. In parallel studies, we tested the capacity of these same MoWs of impairing biofilm formation by several species of viridans streptococci and/or by a mixed bacterial-fungal cell population.

## Materials and methods

### *Candida albicans* strains

The reference strain SC5314 (ATCC MYA-2876) of *C*. *albicans* was employed for the present study. Fungal cultures were maintained by passages onto Sabouraud Dextrose Agar (SDA) plates (OXOID, Milano, Italy) performed at least twice a week. Moreover, the previously described [32] *C*. *albicans* CA1398, carrying the bioluminescence ACT1p-gLUC59 fusion product (BLI *C*. *albicans*) was used. The latter was maintained by biweekly passages in Yeast Peptone Dextrose Agar (YPD). The day before each experiment, fresh *Candida* cultures were seeded onto SDA or YPD plates and incubated at 37°C. After overnight incubation, fungal cells were harvested by a sterile inoculating loop, suspended in phosphate-buffered saline (PBS, EuroClone, Whethereby, UK), washed twice by centrifugation at 3,500 rpm for 10 minutes, counted by Burker’s chamber and suspended at 1 x 10^6^ yeast cells/ml in RPMI 1640 medium supplemented with 10% heat-inactivated fetal bovine serum (hiFBS) (Defined Hyclone, Logan, UT, USA), gentamicin (50 mg/ml; Bio Whittaker, Verviers, Belgium), Ciproxin (2 mg/ml; ICN) and L-glutamine (2 mM; EuroClone, Milan, Italy), from here indicated as “cRPMI”. This fungal cell suspension, composed of yeast-like forms, was used throughout the study to set up all the experiments.

For long-term storage, *C*. *albicans* was maintained as frozen stocks at -80°C, in glycerol solution 20% (v/v) (Incofar S.r.l., Modena, Italy).

### TR146 oral epithelial cell line

The previously described human oral epithelial cell line TR146 [33] was maintained in Dulbecco’s Modified Eagle’s Medium/Ham’s Nutrient Mixture F12 (Sigma, St Louis, MO, USA) supplemented with 15% hiFBS, gentamicin (50 mg/ml), Ciproxin (2 mg/ml) and L-glutamine (2 mM), hereafter referred to as “cF12”. For maintenance, medium was replaced thrice a week. Cells were detached 8 days before the adhesion experiment by adding Trypsin/EDTA solution (2 ml), incubating for 15 min at 37°C with 5% CO_2_ and scraping to detach cells. Then fresh cultures were started in cF12, at a concentration of 10^6^/ml. TR146 cells were used for adhesion assays, cytokines and chemokines determination and in mixed *C*. *albicans*/*S*. *salivarius* biofilm production.

### BV2 microglial cell line

The previously established murine microglial cell line BV2 [34] was maintained in cRPMI. Cells were detached biweekly by vigorous shaking, and fresh cultures were started at a concentration of 5 x 10^5^/ml the day before each experiment.

BV2 cells were employed for the phagocytosis assay.

### Bacterial strains

Bacteria were collected from pharyngeal swabs, seeded on COS plates (Biomerieux, Marcy-L’Etoile, France) and allowed to grow for 24 hours at 35°C. Alpha-haemolytic streptococci colonies were isolated and identification, at species level, was achieved by MALDI-TOF (Biomerieux). The system allowed to identify isolates belonging to the species *S*. *salivarius*, *S*. *sanguinis*, *S*. *parasanguinis*, *S*. *vestibularis*, and *Enterococcus faecalis*, but it could not discriminate between the species *S*. *mitis* and *S*. *oralis*; therefore, the 3 isolates used in the present study were indicated as *S*. *mitis/oralis*. Seventeen isolates were employed: 3 isolates each of the species *S*. *salivarius*, *S*. *mitis/oralis*, *S*. *sanguinis*, *S*. *parasanguinis* and *E*. *faecalis* and 2 isolates of the species *S*. *vestibularis*.

For long-term storage, bacteria were maintained as frozen stocks at -80°C, in Tryptic Soy Broth (TSB, Difco Laboratories, Detroit, MI, USA) with glycerol 5% (v/v).

The day before each experiment, 0.2 ml of bacteria from frozen stocks were seeded in 4 ml of TSB and incubated at 37°C. After overnight incubation, 0.1 ml of bacterial suspensions were placed in 96-well plates and counted by measuring optical density (OD) at 595 nm wavelength, by means of a plate reader (Tecan Sunrise, Austria). The bacterial concentration was calculated by interpolating OD values to a McFarland standard curve [35]. Bacteria were then centrifuged and suspended in Brain Heart Infusion (BHI) broth (Liofilchem S.r.l., Italy) to a concentration of 1.5 x 10^8^ CFU/ml.

### Mouthwashes

Six commercial Mouthwashes (MoWs), operationally indicated with Arabic numbers, were employed. These mouthwashes are the same already employed in a previous study conducted by our group [16]. MoW 1 (CURASEPT 0.20, Curadent Healthcare S.p.A., Saronno (VA), Italy), MoW 2 (DENTOSAN COLLUTORIO, Recordati S.p.A., Milano, Italy) and MoW 3 (MERIDOL COLLUTORIO, Gaba-Colgate-Palmolive, Świdnica, Poland) contained 0.2% chlorhexidine digluconate (CHX). MoW 7 (PARODONTAX, GlaxoSmithKline, Brentford, UK) contained 0.06% CHX and 250 ppm F- sodium fluoride. MoW 4 (ELMEX SENSITIVE PROFESSIONAL, Gaba, Therwil, Switzerland) and MoW 5 (LISTERINE TOTAL CARE ZERO, Johnson&Johnson, Maidenhead, UK) included in their formulation fluorine-containing molecules and essential oils respectively. Two additional commercial mouthwashes, with different formulations, were employed in focused experiments: MoW 8 (ORAL B, Procter & Gamble, U.K.), containing 0.05% cetylpyridinium chloride (CPC) and 0.05% sodium fluoride and MoW 9 (PlaKKontrol Protezione Totale, IDECO, Bolzano, Italy), containing Triclosan and sodium fluoride. Details on MoWs composition are available in S1 Table.

All the MoWs selected for the present study were ethanol-free.

Operationally, the contact times of 5 and 15 minutes between *Candida* and the different MoWs have been chosen in order to mimic the everyday life employment of MoWs. Indeed, manufacturers recommend to avoid eating and drinking for at least 30 minutes after rinsing with MoWs, since the active molecules bind to the teeth and mucosal surfaces and are released gradually over time. Such property, known as substantivity, has been demonstrated for CHX, a molecule widely employed in MoWs formulations. This molecule, besides its direct antibacterial and antifungal action, can bind to surrounding tissues and then be released slowly, over extended periods of time [10,36]. Our previous study has shown that at the *Candida*:MoW 1:2 dilution (i.e., the same employed for the present study) there are no differences on biofilm formation and *Candida* metabolic activity between 1 and 5 minutes contact time with the MoWs; similarly, no differences have been observed between 15 and 30 minutes contact time [16,18]. Therefore, the 5 minutes contact time has been chosen to describe the immediate MoWs effect on *Candida*, whereas the 15 minutes contact time has been chosen to provide information on MoWs substantivity.

### *In vitro* adhesion assays

Adhesion of *Candida* yeast cells was assessed both on abiotic surfaces and to oral epithelial cells.

Adhesion to abiotic surfaces: BLI *C*. *albicans* cells were harvested by a sterile inoculating loop, suspended in PBS, washed twice by centrifugation at 3,500 rpm for 10 minutes, counted by Burker’s chamber and suspended at 2 x 10^6^ yeast cells/ml in PBS. Then, 0.5 ml of *Candida* suspension were added to Eppendorf tubes containing 0.5 ml of PBS or 0.5 ml of the different MoWs, and incubated at 37°C with 5% CO_2_ for 5 or 15 minutes. MoWs were then removed by spinning twice all the tubes at 4,500 rpm for 8 minutes, and by replacing the supernatants every time with PBS. After the last wash, each pellet was suspended in cF12 in order to have a final concentration of 1 x 10^6^ fungal cells/ml. One hundred microliters of these fungal suspensions were added (each condition in triplicate) to black 96-well microtiter plates with a transparent bottom (Perkin Elmer Life Sciences) and incubated for 1 hour at 37°C. Then, each well was gently washed (3 times with 200 μl of PBS) to remove the non-adhered fungal cells. Finally, according to a previously described procedure [32], 2 μM coelenterazine (SynChem, Ohm, Germany) in luciferase assay buffer (LA buffer) were added to each well; the bioluminescence was read immediately with a luminometer (Victor X Light, Perkin Elmer Life Sciences) and the signal was expressed as relative luminescence units (RLU). According to the data by Enjalbert and coworkers [32], bioluminescence sensitivity corresponds to 1,000 gLUC59-expressing *C*. *albicans* cells. After reading, the adhered fungal cells were also evaluated by colony forming units counts (CFU). As detailed elsewhere [37], the LA buffer was removed from each well, 100 μl of Trypsin/EDTA solution were added to each well, then plates were incubated for 15 min at 37°C plus 5% CO_2_ to detach cells. Hence, fungal suspensions were seeded onto SDA plates, which were placed at 37°C for 24 hours. The CFU were then evaluated.

Adhesion to oral epithelial cells: Two-hundred microliters of TR146 human epithelial cells (1 x 10^5^ cells/ml) were seeded into black 96-well microtiter plates with a transparent bottom (Perkin Elmer Life Sciences). The plate was incubated for 3 days at 37°C in order to allow the formation of a monolayer of epithelial cells homogeneously distributed on the bottom of each well. After 3 days, the BLI *C*. *albicans* cells were harvested and the experiment was carried out as detailed above. After reading, the adhered fungal cells were evaluated also by colony forming units counts (CFU), by the same procedure described above for the adhesion to abiotic surfaces.

### *Candida* viability/growth assay

BLI *C*. *albicans* cells (4 x 10^5^/ml) were pre-treated with MoWs or with PBS for 5 and 15 minutes. Then, fungal suspensions were washed twice with PBS to remove MoWs and suspended in 1 ml of PBS. Then, 100 μl of each fungal suspension were seeded onto SDA plates. CFU counts were evaluated after 24 hours of incubation at 37°C.

### Cytokines and chemokines

Two-hundred microliters of TR146 human epithelial cells (1 x 10^5^ cells/ml) were seeded into 96-well microtiter plates (Costar 3595, Corning, NY, USA) and incubated for 3 days at 37°C in order to allow the formation of a homogeneously distributed monolayer of epithelial cells on the bottom of each well. After 3 days, 0.5 ml of *Candida* suspension (2 x 10^6^ yeast cells/ml in PBS) were added to Eppendorf tubes containing 0.5 ml of PBS or 0.5 ml of the different MoWs, and incubated at 37°C with 5% CO_2_ for 5 or 15 minutes. MoWs were then removed by spinning twice all the tubes at 4,500 rpm for 8 minutes, and by replacing the supernatants every time with PBS. After the last wash, each pellet was suspended in cF12 in order to have a final concentration of 1 x 10^6^ fungal cells/ml. One hundred microliters of these suspensions were added to the TR146-containing wells of the 96-well microtiter plate and incubated for 24 hours at 37°C. As positive control, TR-146 cells were stimulated with 1 μg/ml of LPS. As negative controls, wells containing TR-146 cells, which did not undergo infection with *Candida* or stimulation with LPS, were included. Each condition was tested in triplicate. Then, supernatants were collected and cytokines/chemokines levels were assessed by means of Quantibody Human Inflammation Array 1 (Ray Biotech Inc., Norcross, GA, USA), an antibody microarray system already employed for cytokines/chemokines determinations in several biological fluids [38,39]. With this array the following cytokines/chemokines could be quantitatively detected: IL-1α, IL-1β, Interleukin-4 (IL-4), IL-6, IL-8, Interleukin-10 (IL-10), Interleukin-13 (IL-13), Monocyte Chemoattractant Protein-1 (MCP-1), Interferon-γ (IFN-γ), Tumor Necrosis Factor-α (TNF-α). The supernatants were used undiluted and the assay was conducted according to the Manufacturer’s instructions. The fluorescent signal from the slides was read by using a ScanArray Gx scanner (Perkin-Elmer, Cambridge, UK). Images generated were saved as TIFF files and quantified with the ScanArrayExpress software, provided by Perkin-Elmer. Since no specific indications on cut-offs were provided, all the cytokine/chemokine levels which, interpolated to their respective calibration curves, resulted below 0 pg/ml, were considered as negative.

### *In vitro* phagocytosis assay

*C*. *albicans* SC5314 cells were harvested by a sterile inoculating loop, suspended in PBS, washed twice by centrifugation at 3,500 rpm for 10 minutes, counted by Burker’s chamber and resuspended at 2 x 10^6^ yeast cells/ml in PBS. Then, 0.5 ml of *Candida* suspension were added to Eppendorf tubes containing 0.5 ml of PBS or 0.5 ml of the different MoWs, and then incubated at 37°C with 5% CO_2_ for 5 or 15 minutes. MoWs were then removed by spinning all the tubes twice at 4,500 rpm for 8 minutes, and by replacing the supernatant every time with PBS. After the last wash, each pellet was suspended in 100 μl of FITC fluorescent dye (Sigma), previously diluted 1:10 with PBS, and incubated for 20 minutes. FITC was then removed by spinning all the tubes three times at 4,500 rpm for 8 minutes and by replacing the supernatant every time with PBS. After the last washing, each pellet was suspended in 240 μl of cRPMI in order to have working strength suspensions of 4 x 10^6^ fungal cells/ml.

In the meantime, BV2 cells were seeded into Lab-Tek II chamber slides wells (Nalge Nunc International, Naperville, IL, USA). In order to strengthen adhesion of BV2 cells to the glass bottom of the wells, the latter were pretreated with poly-L-lysine (Sigma-Aldrich; 10 μg/well), for 30 minutes, and washed twice with PBS. BV2 cells (2 x 10^6^ cells/ml, 100 μl/well) were then seeded and incubated for 30 minutes at 37°C with 5% CO_2_. BV2 cells were thus infected by adding 100 μl of *Candida* suspension, the effector-target (E:T) ratio being 1:2. Then, 10 μl/well of LysoTracker Red DND-99 fluorescent dye (Molecular Probes, Life Technologies, Eugene, Oregon, USA), at 5 mM working strength concentration, were added. The chamber slides were then incubated for 1.5 hours at 37°C with 5% CO_2_. Thirty minutes before the end of incubation, an additional volume of 10 μl of LysoTracker Red DND-99 fluorescent dye were added to each well. Fifteen minutes before the end of incubation, 40 μl of Uvitex 2B fluorescent dye (Polysciences, Inc, PA, USA) were added to each well of the chamber slide.

At the end of the incubation, all the wells were washed twice with warm cRPMI (5 minutes each) and fixed for 30 minutes with 4% paraformaldehyde (PFA) (Sigma-Aldrich) in PBS at +4°C, protected from light. PFA was then removed by means of a double wash with cold PBS (10 minutes each). The chamber slide was then unset, by removing the plastic walls of the wells and the slide surface was treated with ProLong Gold Antifade Reagent (Molecular Probes, Invitrogen, St. Louis, Mo, USA) in order to suppress the photobleaching effect and to preserve the signals of fluorescently labeled target molecules. The fluorescence of phagocytosed and non-phagocytosed fungi was visualized by means of epifluorescence microscopy Nikon Eclypse 90i (Nikon Instruments, Tokyo, Japan) equipped with different filters for reading different fluorescent signals. For clarity, FITC fluorescent dye stained in green all *Candida* cells and its signal was read by FITC filter. LysoTracker Red DND-99 fluorescent dye stained in red the *Candida* cells inside acidic phagolysosomes and its signal was read by TRITC filter. Uvitex 2B fluorescent dye stained in blue all the *Candida* cells that had not been phagocytosed and its signal was read by DAPI filter. The phagocytosis percentage was then calculated by counting the cells containing one or more *Candida* on a total of 200 phagocytic cells scored. The phagocytosis index was calculated by counting the total number of phagocytosed *Candida* (including those inside acidic phagolysosomes) on a total of at least 200 *Candida*-containing phagocytic cells. The acidic phagolysosomes percentage was calculated on a total of at least 200 *Candida*-containing phagocytic cells by counting *Candida* cells inside acidic phagolysosomes on the total number of phagocytosed *Candida* cells.

### Biofilm production by oral streptococci and *E*. *faecalis*

Bacterial biofilm production was assessed for each individual *Streptococcus* isolate. Biofilm was allowed to form on flat-bottom 96-well plates (Corning Incorporated, NY, USA), according to the protocol described by Stepanović and coworkers, with minor modifications [40]. Briefly, 1.8 ml of bacteria cell suspensions (1.2 x 10^7^ CFU/ml) in BHI broth were added to 1.8 ml of each MoW or PBS and incubated at 37°C for 1 minute. Then, MoWs were removed by spinning at 4,500 rpm for 8 minutes and by suspending the pellets in PBS. After the last washing step, pellets were suspended in BHI broth to a working strength concentration of 1.5 x 10^7^ CFU/ml, seeded in the wells of a 96-well plate (each condition in triplicate) and incubated at 37°C with 5% CO_2_ for 48 hours. Finally, to measure biofilm formation, Crystal Violet (CV) assay was performed. The OD was read at 570 nm wavelength, by means of a plate reader (Tecan Sunrise, Austria). The capacity to form BF of MoWs-treated bacteria was expressed as OD percentage, as compared to the OD of the PBS-treated counterpart, which was considered as 100%.

### *C*. *albicans* biofilm production upon co-culture with MoWs-treated *S*. *salivarius*

An isolate belonging to the species *S*. *salivarius* (considered a “pro-cariogenic” species) was selected to study the influence (if any) of MoWs-pretreated streptococci on the capacity to form biofilm by *C*. *albicans*. This particular isolate was chosen because of its susceptibility to most of the MoWs employed (see below).

Two-hundred microliters of TR146 human epithelial cells (1 x 10^5^ cells/ml) were seeded into black 96-well microtiter plates and incubated for 3 days at 37°C, in order to allow the formation of a monolayer of epithelial cells homogeneously distributed on the bottom of each well. After 3 days, 600 microliters of *S*. *salivarius* isolate 12 cell suspensions (1.5 x 10^7^ CFU/ml) in BHI broth were added to 600 μl of each MoW or PBS and incubated at 37°C for 1 minute. Then, MoWs were removed by spinning at 4,500 rpm for 8 minutes and by suspending the pellets in PBS. After the last washing step, pellets were suspended in cF12 medium, seeded (each condition in triplicate) together with BLI *C*. *albicans* (1 x 10^6^/ml, 100 μl/well) in the TR146-containing wells of the 96-well plate described above and incubated at 37°C with 5% CO_2_ for 24 hours. After removal of the suspensions containing the non-adhered fungal cells, the wells were washed 3 times with 200 μl of PBS. Finally, 2 μM coelenterazine in LA buffer was added to each well and the bioluminescent signal was read immediately with a luminometer (Victor X Light, Perkin Elmer Life Sciences). After reading, the supernatants from each well were thrown, substituted with the same volume of fresh cF12 medium and incubated for further 24 hours. After this time, all the wells were washed 3 times (as described above), 2 μM coelenterazione in LA buffer was added to each well and the bioluminescent signal was read immediately with the luminometer.

### Statistical analysis

All the data contained in the graphs are expressed as average values ± standard errors of at least 3 independent experiments. Analysis of variance (ANOVA) and Bonferroni post-hoc test were carried out by IBM SPSS Statistics 23 to assess overall differences amongst MoWs-treated groups in relation to control groups. Statistical significance was set at *p*<0.05.

## Results

### Adhesion of MoWs-treated *C*. *albicans* to abiotic surface and epithelial cells

**Fig 1:**
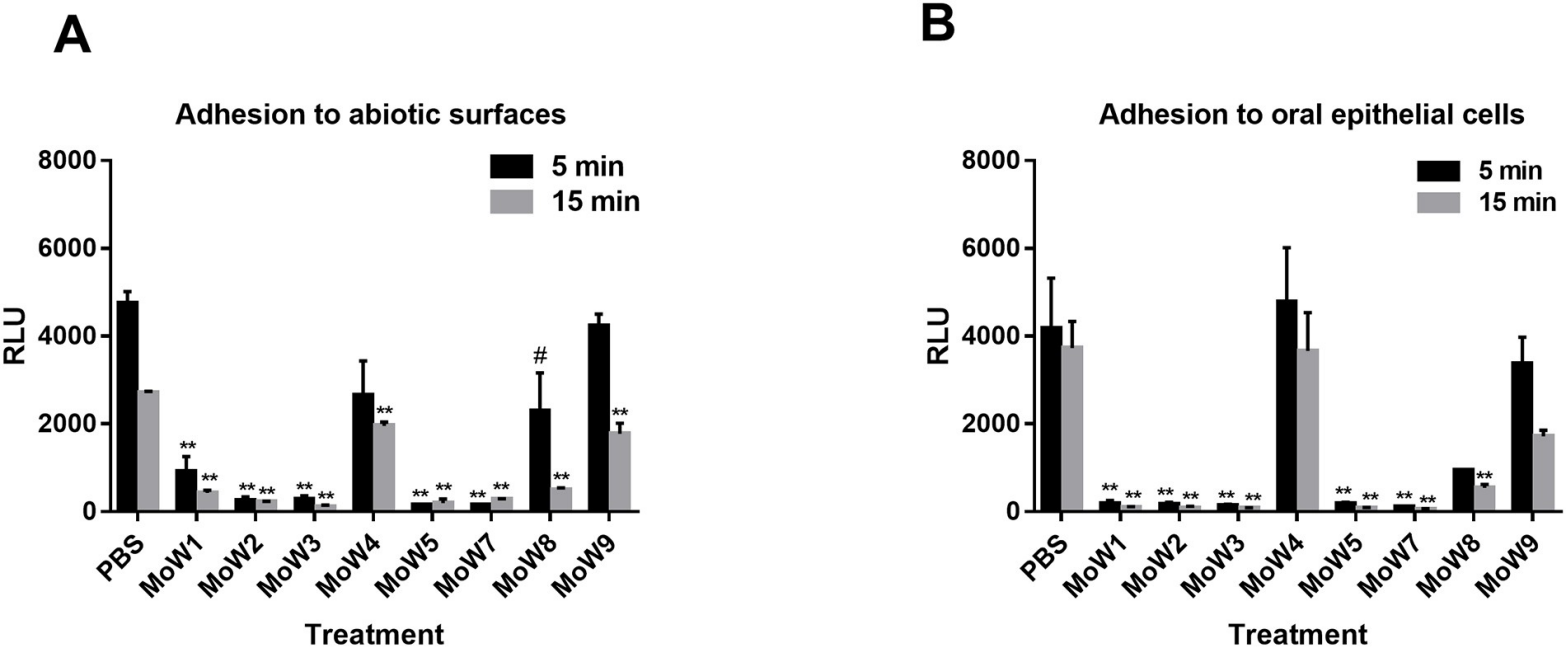
Adhesion of MoWs-treated BLI *C*. *albicans* to plastic and epithelial cells. Yeast cells, treated for 5 minutes (black columns) or 15 minutes (grey columns) with MoWs or PBS (as a control), were washed, suspended in cF12 and seeded (1 x 10^5^ cells/well) in 96-well plates where they were let adhere for 1 hour at 37°C. Then, the adhesion to abiotic surfaces (plastic bottom of the well plates—**A**) and adhesion to biotic surfaces (TR146-covered bottom of the well plates—**B**) were assessed by bioluminescence. Signals collected are expressed as relative luminescence units (RLU). The results are mean values ± standard errors of triplicates. ^#^p< 0.05 or **p< 0.001 indicate significant differences between MoWs-treated vs PBS-treated BLI *C*. *albicans*. MoW1: Curasept 0.20; MoW2: Dentosan Collutorio; MoW3: Meridol Collutorio; MoW4: Elmex Sensitive Professional; MoW5: Listerine Total Care Zero; MoW7: Parodontax; MoW8: Oral B; MoW9: PlaKKontrol Protezione Totale.

In order to evaluate the effects of the MoWs on *C*. *albicans* capacity to adhere to abiotic (plastic) and biotic (TR146 epithelial cells) surfaces, BLI *C*. *albicans* was employed. In particular, fungal cells treated either with MoWs or PBS (for 5 or 15 minutes) were seeded into a 96-well plate and were allowed to adhere for 1 hour at 37°C, before washing and bioluminescence measurement. As expected, *Candida* cells treated with PBS returned the highest bioluminescent signals, indicating the capacity of the fungus *per se* to adhere to the plastic of the microwells. When *Candida* was treated with MoWs 1, 2, 3, 5, 7 and 8, little or no bioluminescent signals were detectable, indicating a failure of the fungus to adhere to the plastic. These results were consistent, irrespective of the MoWs incubation times; statistical analysis showed that bioluminescent readings from the wells containing MoWs-treated *Candida* were significantly lower than readings from PBS-treated *Candida*, used as negative control. MoW 4- and MoW 9-treated *Candida* returned a bioluminescent signal higher than those from the other MoWs. Yet, after 15 minutes of incubation such signal was significantly lower than that recorded from PBS-treated *Candida*, indicating that, tough less effective than the others, MoWs 4 and 9 were still capable to significantly impair *Candida* adhesion to the wells (Fig 1A).

Adhesion of MoWs-treated *C*. *albicans* to TR146 cells was significantly lower than that of PBS-treated *Candida*, for all but MoWs 4 and 9. *Candida* cells treated with MoWs 1, 2, 3, 5, 7 and 8 returned little or no bioluminescent signals, indicating an almost complete failure of the fungus to adhere to the oral epithelial cells, after MoWs treatment. On the contrary, MoW 4- and MoW 9-treated *Candida* returned a bioluminescent signal, comparable to that collected from PBS-treated *Candida*, indicating that adhesion to the epithelial cells remained substantially not affected by these MoWs (Fig 1B). CFU counts of adhered fungal cells confirmed the bioluminescence results: > 300 CFU/ml for PBS-, MoW 4- and MoW 9-treated *Candida* and < 30 CFU/ml for MoW 1-, MoW 2-, MoW 3-, MoW 5-, MoW 7- and Mow 8- treated *Candida*, after 15 min both on abiotic and biotic surfaces.

In order to verify if adhesion impairment by MoWs was due also to a decrease in fungal cells viability, we evaluated the CFU counts of fungal cells pre-incubated for 5 or 15 minutes with MoWs. Our results showed that growth of *Candida* pre-treated with MoWs 2, 3, 5, 7 and 8 (irrespective of the contact times) was almost completely inhibited (< 30 CFU). Differently, from undiluted supernatants of MoW 4- and PBS-treated *Candida*, more than 300 CFU could be counted, regardless of the contact times. From fungal suspensions pre-treated with MoW 1 and MoW 9, 115 ± 5.6 and 112 ± 4.2 and 245 ± 7.4 and 230 ± 2.5 CFU respectively could be counted after 5 or 15 minutes contact time.

### Cytokines and chemokines secretion by TR146 oral epithelial cells incubated with MoWs-treated *C*. *albicans*

**Fig 2:**
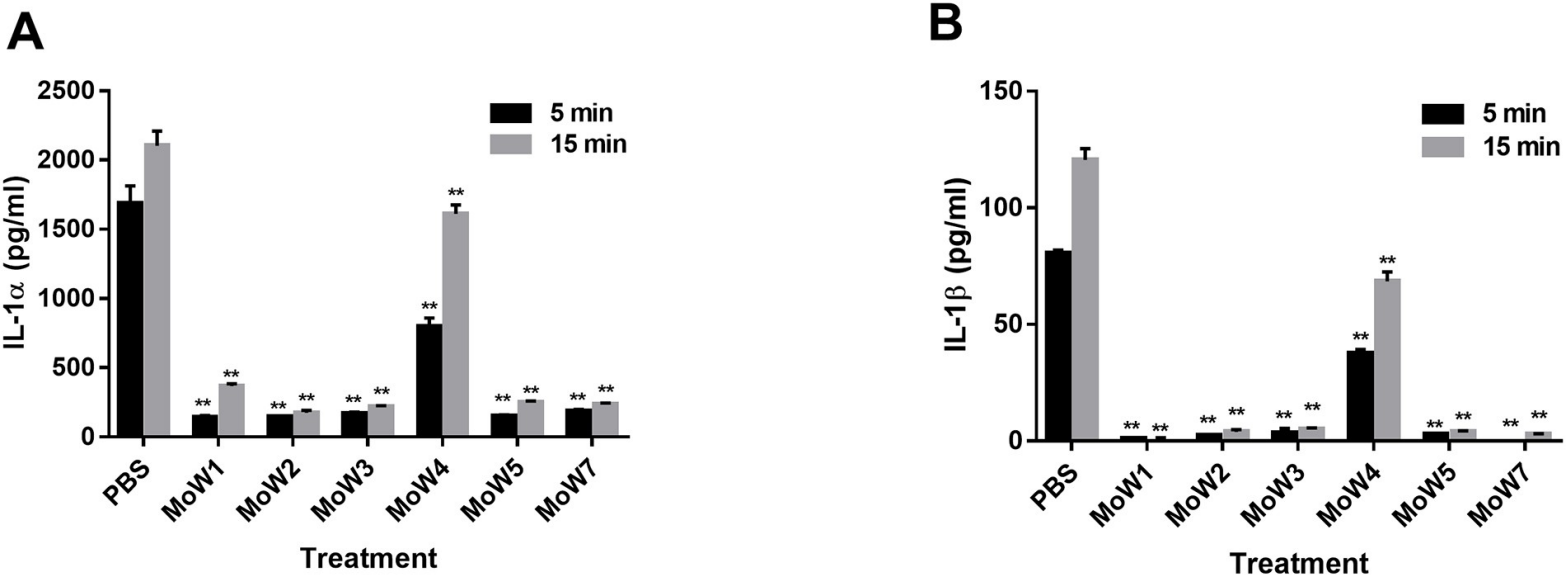
Induction of proinflammatory cytokines secretion by TR146 cells infected with MoWs-treated *C*. *albicans*. Yeast cells, treated for 5 minutes (black columns) or 15 minutes (grey columns) with MoWs or PBS (as negative control), were washed, suspended in cF12 and seeded (1 x 10^5^ cells/well) in 96-well plates containing a 3 days old monolayer of TR146 cells. After 24 hours incubation at 37°C, supernatants were collected and cytokines/chemokines levels were assessed by an antibody microarray. Levels of IL-1α (**A**) and IL-1β (**B**) are shown. The results are mean values ± standard errors of triplicates. **p< 0.001 indicate significant differences between MoWs-treated vs PBS-treated *Candida*. MoW1: Curasept 0.20; MoW2: Dentosan Collutorio; MoW3: Meridol Collutorio; MoW4: Elmex Sensitive Professional; MoW5: Listerine Total Care Zero; MoW7: Parodontax.

*C*. *albicans*, pretreated with MoWs or PBS, was used to infect monolayers of TR146 oral epithelial cells; 24 h later, the capacity of *Candida* to induce a secretory response was evaluated. By using an antibody microarray assay, the supernatants were screened for levels of IL-1α, IL-1β, IL-4, IL-6, IL-8, IL-10, IL-13, MCP-1, IFN- γ and TNF-α. *Candida* cells treated with PBS were capable to induce the secretion of both IL-1α and IL-1β (but not of the other cytokines and chemokines) by TR146 oral epithelial cells. When TR146 cells were infected with MoWs-pretreated *Candida*, a significant reduction in the secretion of both IL-1α (Fig 2A) and IL-1β (Fig 2B) was observed with respect to controls, namely TR146 cells infected with PBS-pretreated *Candida*. The most pronounced reduction was observed upon *Candida* pretreatment with MoWs 1, 2, 3, 5 and 7, irrespective of the 5 or 15 minutes contact time. Also, *Candida* pretreatment with MoW 4 significantly impaired fungal capacity to elicit IL-1α and IL-1β, but to a lesser extent (Fig 2A and 2B). None of the other cytokines/chemokines assessed by protein array were detectable at appreciable levels in the same culture supernatants. As expected, LPS stimulation induced secretion of IL-1α, IL-1β and TNF-α. The values of pg/ml obtained after LPS stimulation, were 1004.7 pg/ml ± 35.8 for IL-1α and 53 pg/ml ± 5.2 for IL-1β.

### Susceptibility to phagocytosis of MoWs-treated *C*. *albicans*

**Fig 3:**
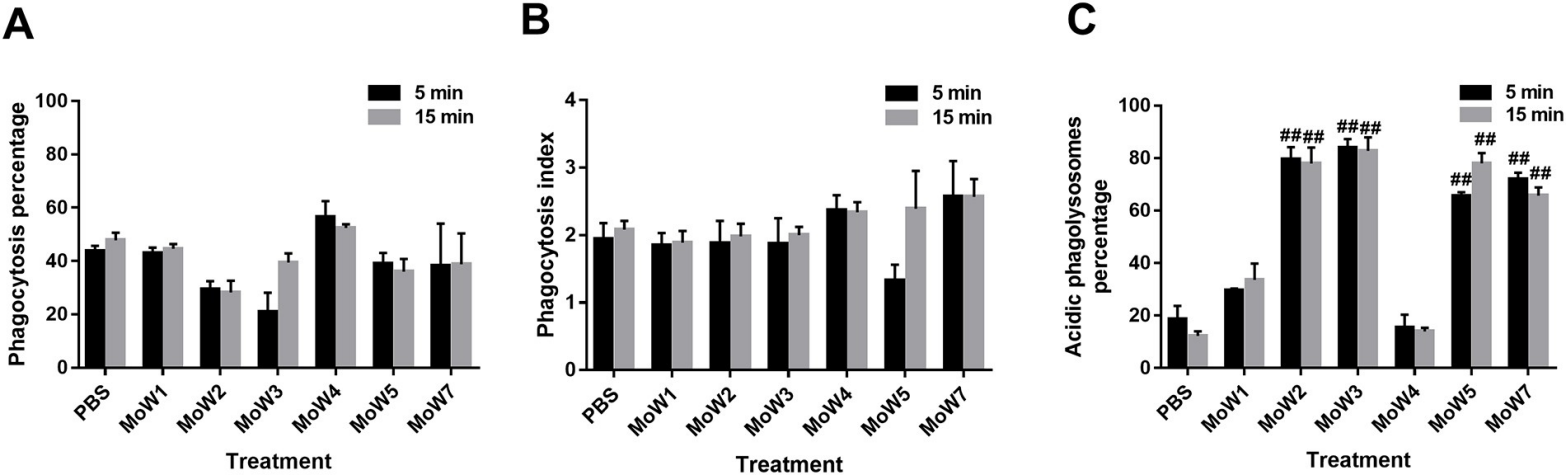
Effects of the different MoWs on *C*. *albicans* susceptibility to phagocytic cells. Yeast cells, treated for 5 minutes (black columns) or 15 minutes (grey columns) with MoWs or PBS, as a control, were washed, suspended in cRPMI, fluorescently labelled and seeded (2 x 10^5^ cells/well) in chamber-slides containing BV2 phagocytic cells (1 x 10^5^ cells/well; E:T ratio = 1:2). After 1.5 hours incubation at 37°C, slides were fixed and fluorescent signal was acquired. The following parameters were then calculated: phagocytosis percentage **(A)**, phagocytosis index **(B)** and acidic phagolysosomes percentage **(C)**. The results are mean values ± standard errors of at least triplicate samples. ^##^p< 0.001 indicate significant differences between MoWs-treated vs PBS-treated *Candida*. MoW1: Curasept 0.20; MoW2: Dentosan Collutorio; MoW3: Meridol Collutorio; MoW4: Elmex Sensitive Professional; MoW5: Listerine Total Care Zero; MoW7: Parodontax.

Susceptibility to phagocytosis of MoWs-treated *Candida* cells was assessed by means of an *in vitro* assay, which uses a previously established murine microglial cell line BV2, known to efficiently ingest and intracellularly kill *Candida* [41]. Thus, PBS- or MoWs-pretreated *C*. *albicans* cells (5 or 15 minutes) were exposed to BV2 cells for 1.5 hours; then, phagocytosis percentage, phagocytosis index and acidic phagolysosomes percentage were calculated. By ANOVA test, the differences in phagocytosis percentage amongst the MoWs-treated samples and the PBS-treated controls never reached the level of significance (always p>0.05), irrespective of the contact time (Fig 3A).

Similarly, the differences in phagocytosis index amongst the MoWs treated samples and the PBS-treated controls never reached the level of significance (always p>0.05) (Fig 3B). Differently, when the acidic phagolysosomes percentage was assessed, two trends were detected. Specifically, *Candida* cells treated with MoWs 1 and 4 returned acidic phagolysosomes percentages not statistically different from those of the PBS-pretreated *Candida*, as assessed by ANOVA test (p>0.05). On the contrary, *Candida* cells treated with MoWs 2, 3, 5 and 7 returned an acidic phagolysosome percentage much higher than the controls’. By ANOVA test, such values were significantly higher than those of the control samples (p<0.05). Such results were independent from the 5 or 15 minutes contact times (Fig 3C).

**Fig 4:**
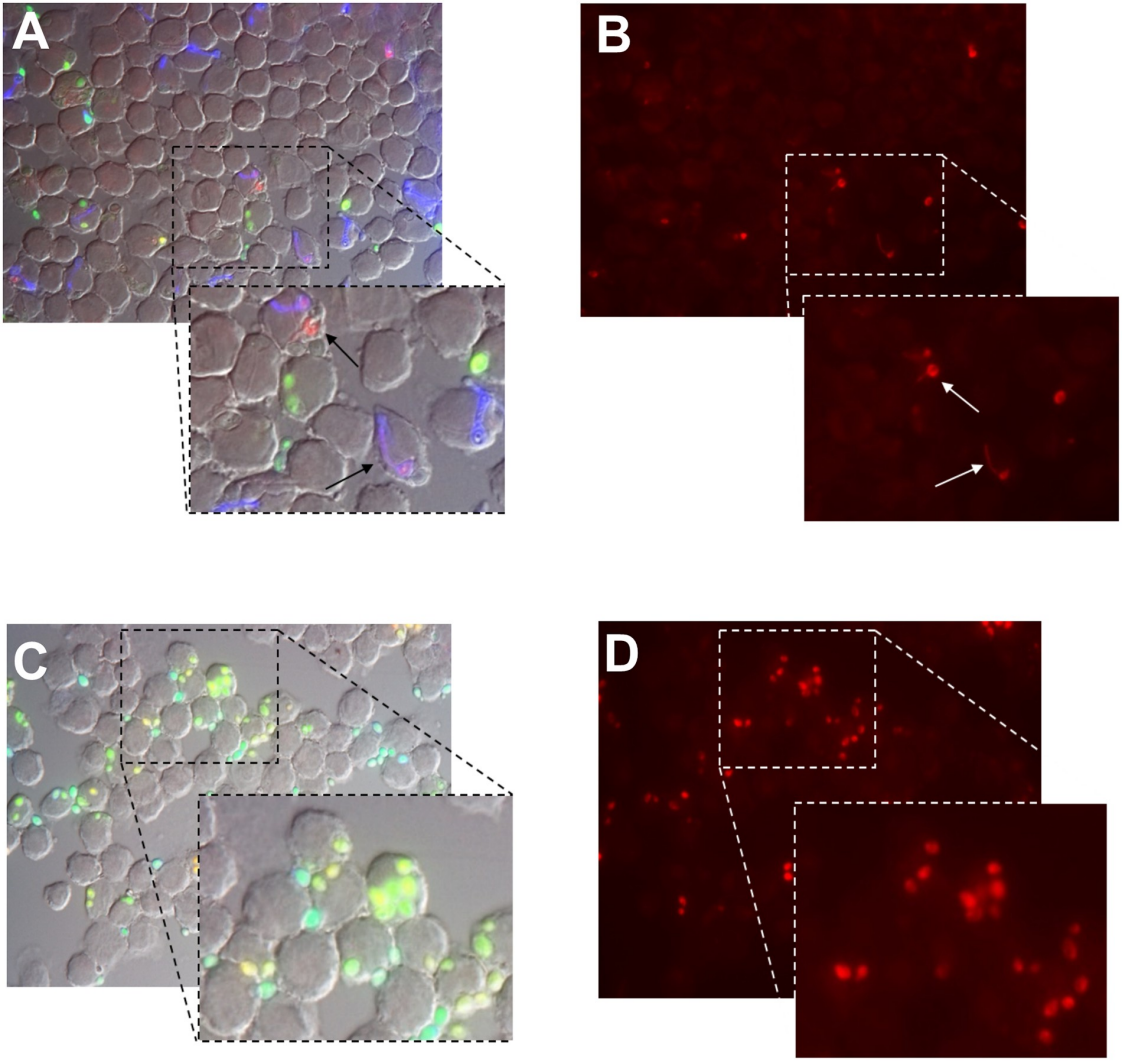
Morphology of *C*. *albicans* treated with the different MoWs and then exposed to phagocytic cells. Representative fluorescent microscopy images of yeast cells, treated for 5 or 15 minutes with MoWs or PBS, as a control, washed, suspended in cRPMI, fluorescently labelled and seeded (2 x 10^5^ cells/well) in chamber-slides containing BV2 phagocytic cells (1 x 10^5^ cells/well; E:T ratio = 1:2). After 1.5 hours incubation at 37°C, slides were fixed and fluorescent signal was acquired. *C*. *albicans* cells treated with PBS, MoW 1 or MoW 4 retain their ability to undergo dimorphic transition, even when phagocytized by macrophages (representative image in panel **A** of MoW 4 –black arrows), therefore, only little numbers of red spots (representative image in panel **B** of MoW 4—white arrows) are visible. *C*. *albicans* cells treated with MoWs 2, 3, 5 and 7, are inhibited in their capacity to undergo dimorphic transition and they retain their yeast morphology (representative image in panel **C** of MoW 2), therefore most of them are localized inside acidic phagolysosomes (representative image in panel **D** of MoW 2) where they are likely inactivated. The different colours displayed by fungal cells in panels A and C indicate the fungi simply phagocytized (green *Candida*, stained with FITC), the fungi phagocytized and associated with acidic phagolysosomes (red *Candida*, stained with the acidotropic LysoTracker DND-99 dye), or the fungi not phagocytized by BV2 cells (blue *Candida*, stained with Uvitex 2B). Panels B and D show the same images of panels A and C respectively, using the TRITC filter, which highlights only the *Candida* cells (stained in red with LysoTracker DND-99 dye) localized within the acidic phagolysosomes. Every condition was assessed in at least 3 different experiments. MoW1: Curasept 0.20; MoW2: Dentosan Collutorio; MoW3: Meridol Collutorio; MoW4: Elmex Sensitive Professional; MoW5: Listerine Total Care Zero; MoW7: Parodontax.

Morphological analysis by microphotographs revealed that *Candida* cells, exposed to MoW 1, MoW 4 or PBS could develop hyphae, (Fig 4A) and, as shown by the TRITC filter which reads the red fluorescence of the Lyso-Tracker dye, only a few red spots were detectable (Fig 4B–white arrows). On the contrary, in samples treated with MoWs 2, 3, 5 and 7, no hyphal forms were observed (Fig 4C) and, as assessed by TRITC filter, red spots were abundantly observed (Fig 4D).

### Effects of MoWs on biofilm production by streptococci and *E*. *faecalis*

**Fig 5:**
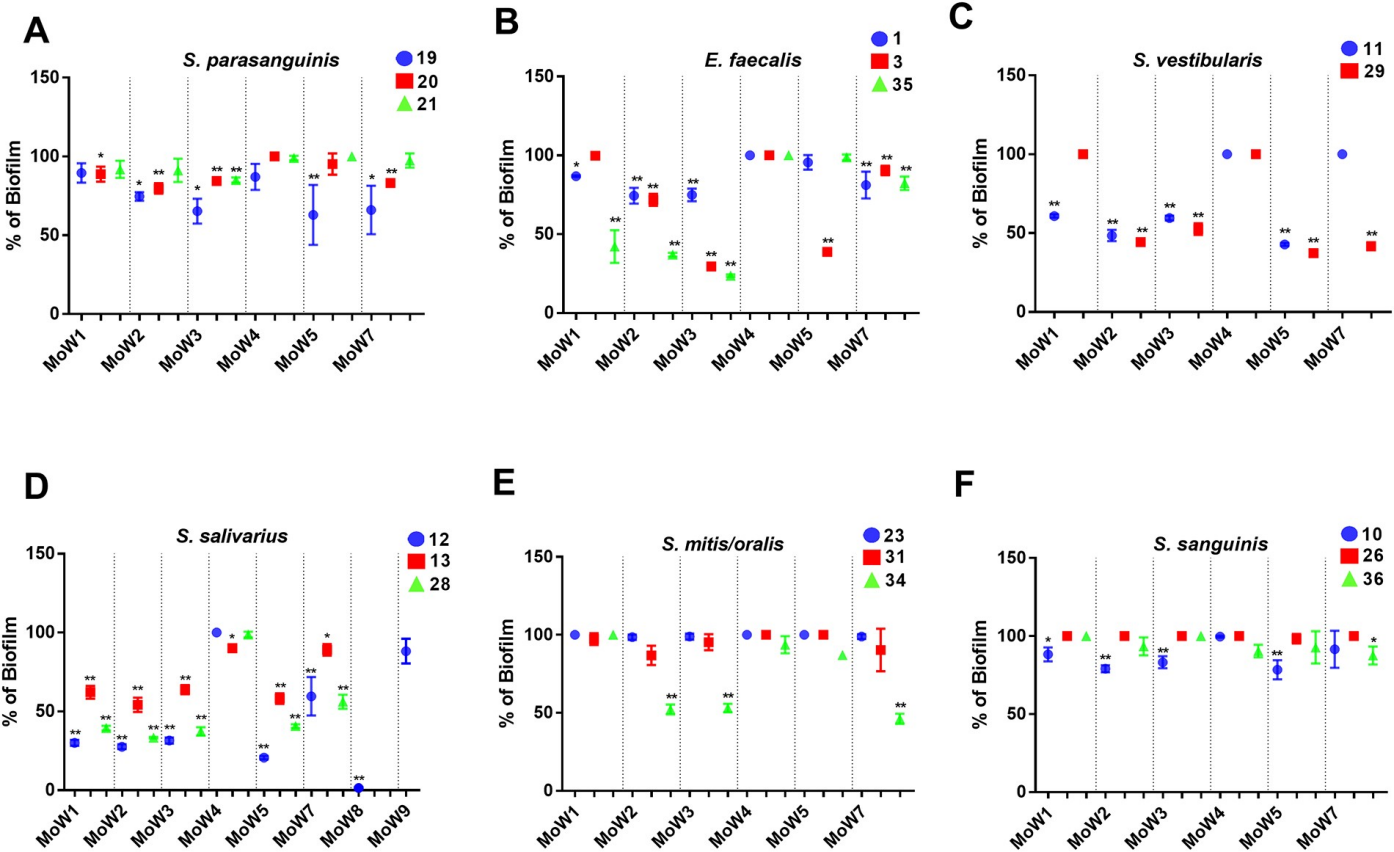
Effects of MoWs on viridans streptococci and *E*. *faecalis* biofilm production. Bacterial isolates, belonging to the species *S*. *parasanguinis* **(A)**, *E*. *faecalis* **(B)**, *S*. *vestibularis* **(C)**, *S*. *salivarius* **(D)**, *S*. *mitis/oralis* **(E)** and *S*. *sanguinis* **(F)** were treated for 1 minute with PBS or with MoWs, before being washed, suspended in BHI broth, seeded (1.5 x 10^7^ CFU/ml) in 96-well plates and incubated at 37°C for 48h to allow biofilm formation. Biofilm biomass was then assessed by CV assay and the results were expressed as percentage of biofilm production with respect to the 100% biofilm of PBS-treated bacteria. Each result is the mean value ± standard error of triplicate samples. *p< 0.05 or **p< 0.001 indicate significant differences between MoWs-treated vs PBS-treated bacteria. MoW1: Curasept 0.20; MoW2: Dentosan Collutorio; MoW3: Meridol Collutorio; MoW4: Elmex Sensitive Professional; MoW5: Listerine Total Care Zero; MoW7: Parodontax; MoW8: Oral B; MoW9: PlaKKontrol Protezione Totale.

The effects (if any) of the MoWs on the capacity of oral streptococci to produce biofilm were investigated. Isolates from 5 species of oral streptococci and *Enterococcus faecalis* were treated for 1 minute with the same MoWs used for *Candida* and then allowed to form a biofilm. Biofilm formation was assessed after 48 hours incubation. Control groups, treated for 1 minute with PBS, showed that all the isolates were capable to produce biofilm, under the experimental conditions detailed in Material and Methods. However, when pre-treated with the MoWs, differences in the capacity to form biofilm could be observed, amongst species and according to the MoWs employed. Specifically, for *S*. *parasanguinis*, *E*. *faecalis*, *S*. *vestibularis* and *S*. *salivarius* the biofilm production was significantly reduced (even though with slight differences amongst isolates) by the treatment with all the MoWs, but MoW 4. The bacteria incubated with MoW 4 were able to produce a biofilm mass very similar to the controls (Fig 5A to 5D).

Differently, the *S*. *mitis/oralis* and *S*. *sanguinis* isolates were poorly affected by the treatment with any of the MoWs, with the exception of *S*. *mitis/oralis* isolate 34 (that was inhibited by MoWs 2, 3 and 7) and *S*. *sanguinis* isolate 10 (that was inhibited by MoWs 1, 2, 3 and 5) (Fig 5E and 5F).

Moreover, being *S*. *salivarius* isolate 12 particularly susceptible to most of the MoWs, it was chosen to test CPC-containing MoW 8 and triclosan-containing MoW 9. We found that the two MoWs had opposite effects on biofilm formation. As shown in Fig 5, panel D, MoW 8 completely inhibited biofilm formation, whereas the contact with MoW 9 did not have any effect.

### Effects of MoWs on mixed biofilm

**Fig 6:**
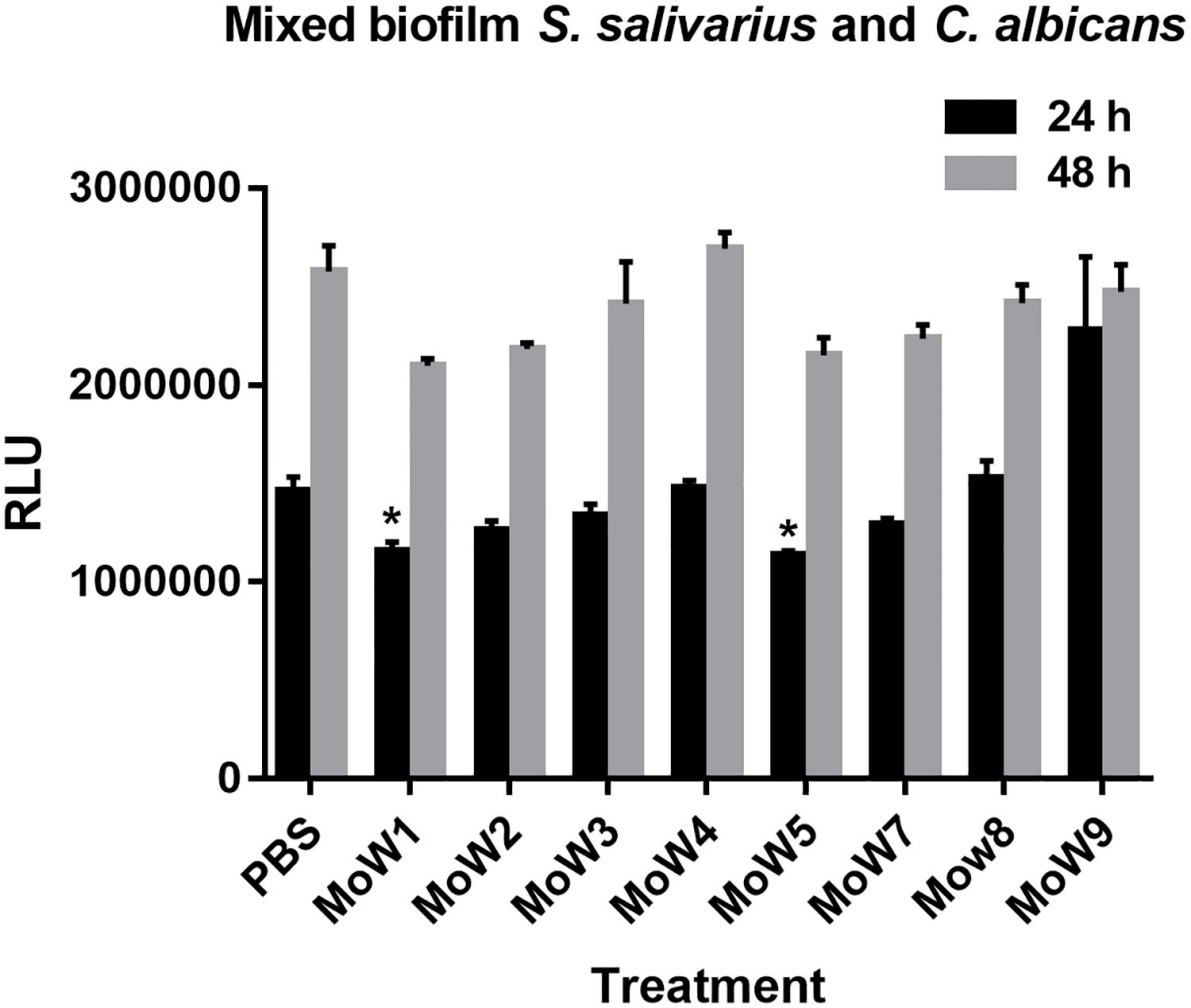
Effect of MoWs-pretreated *S*. *salivarius* on *C*. *albicans* capacity to form biofilm. *S*. *salivarius* isolate 12 was treated for 1 minute with PBS or with MoWs, before being washed, suspended in cF12 and seeded (1.5 x 10^7^ CFU/ml) together with untreated *C*. *albicans* cells (2 x 10^6^/ml) onto a TR146 epithelial cells monolayer. The plates were then incubated at 37°C and BLI *C*. *albicans* biofilm biomass was then assessed by bioluminescence at 24 hours (black columns) and 48 hours (grey columns). Each result is the mean value ± standard error of triplicate samples. *p< 0.05 indicate significant differences between MoWs-treated vs PBS-treated bacteria. MoW1: Curasept 0.20; MoW2: Dentosan Collutorio; MoW3: Meridol Collutorio; MoW4: Elmex Sensitive Professional; MoW5: Listerine Total Care Zero; MoW7: Parodontax; MoW8: Oral B; MoW9: PlaKKontrol Protezione Totale.

As detailed above, amongst the investigated streptococci, *S*. *salivarius* isolates were the most affected by MoWs treatment. Therefore, in order to assess if the MoWs-mediated impairment of *S*. *salivarius* biofilm formation could affect also *C*. *albicans* biofilm formation, *S*. *salivarius* isolate 12 was treated with the different MoWs or PBS for 1 minute and then added, together with untreated bioluminescent *C*. *albicans*, to wells containing 3 day old monolayers of TR146 oral epithelial cells. Plates were then incubated at 37°C and the BLI *C*. *albicans* biofilm formation was assessed, by bioluminescence, after 24h (early biofilm) and 48 hours (mature biofilm). The results, after 24 hours incubation, showed that the treatment of *S*. *salivarius* with MoWs 1 and 5 significantly (p<0.05) impaired *C*. *albicans* biofilm formation at early stages, with respect to *Candida* incubated with PBS-pretreated *S*. *salivarius*. Differently, no effects on *C*. *albicans* early biofilm were observed when *S*. *salivarius* had been pretreated with the other MoWs or with PBS. Bioluminescence measurement after 48 hours showed no effects on *C*. *albicans* biofilm production, irrespective of the fact that *S*. *salivarius* had or had not been pretreated with any of the MoWs (Fig 6).

## Discussion

In a recent study [16], we have shown that several commercial MoWs exert anti-*Candida* activity. The observed differences in yeast cell growth, hyphal formation and biofilm production and persistence have been shown to depend on the MoWs composition and the fungal morphological/functional status. Here, we expand such results by investigating the effects of the same commercial MoWs on other *Candida* virulence factors, such as adhesion to abiotic and biotic surfaces, capacity to elicit pro-inflammatory responses by epithelial cells and susceptibility to phagocytosis and intracellular killing.

*C*. *albicans* capacity to adhere to both abiotic and biotic surfaces is the first stage of fungal invasion [9] and biofilm formation [42]. Here, we show that the treatment with the MoWs significantly impairs *Candida* capacity to adhere to plastic, irrespective of the contact times. Such impairment has been observed with all the MoWs, even though to a lesser extent when using MoWs 4 and 9. Therefore, the MoWs employed in the present study may help prevent oral fungal infections via the impairment of *C*. *albicans* adhesion to abiotic surfaces, frequently present in several types of patients. In addition, MoWs 1, 2, 3, 5, 7 and 8 have shown to impair *Candida* adhesion also to oral epithelial cells. Interestingly, MoWs 4 and 9 only impair (without blocking completely) *Candida* adhesion to plastic, leaving the fungus the ability to adhere to oral epithelial cells. These data suggest that such two MoWs are likely effective only in reducing non-specific forces, such as cell surface hydrophobicity [6–10], while the binding between fungal adhesins and epithelial receptors remains unaffected. Noteworthy, the observed impairment of adhesion mirrors a decrement of viable fungal cells by the direct contact with MoWs, as assessed by bioluminescence sensitivity.

Pretreatment of the fungus with the MoWs alters epithelial cell secretory response to some extent. In particular, IL-1α and IL-1β, secreted at high levels upon infection, significantly decrease in epithelia infected with MoWs-pretreated *Candida*. To our opinion, this result suggests that MoWs may play a relevant role in modulating *Candida* virulence. Epithelia, which cover all the mucosal surfaces of human body, must be able to discriminate between the invasive (pathogenic) and the commensal (harmless) forms of *Candida*, in order to prevent fungal invasion [12]. The reduced secretion of the pro-inflammatory cytokines IL-1α and IL-1β when *Candida* is pre-exposed to MoWs, indicates that under these conditions epithelial cells do not sense *Candida* as a threat, suggesting that fungal virulence potential has been attenuated. Differently, significant production of IL-1α and IL-1β occurs when epithelial cells are infected with PBS-treated fungi (control group); thus, under these conditions, the fungus retains its pathogenic potential, maintaining its capacity to adhere and to invade host tissues, as it may often be perceived at the mucosal level.

By a previously established *in vitro* model, which makes use of the murine microglial cell line BV2, as a prototype of tissue macrophages endowed with potent phagocytic activity and intra-cellular killing against *Candida* [34,41], here we show that the exposure to MoWs affects *Candida* susceptibility to such host-mediated first line defense. We show that, irrespective of the PBS or MoWs pretreatment, approximately 50% of the BV2 cells are able to ingest *Candida* cells, with a phagocytosis percentage and a phagocytosis index that remain substantially unchanged among groups. These data imply that MoWs do not alter the way phagocytes perceive the fungus in their surrounding environment, since their phagocytic activity remains almost unaffected. Differently, assessment of acidic phagolysosomes percentage highlights significant differences, depending upon the MoWs used. When *Candida* cells are pretreated with MoWs 1 and 4, the acidic phagolysosomes percentages remain substantially unchanged and similar to those of PBS-pretreated *Candida*. Morphological observations clearly show that microglial cells that have ingested *Candida* pretreated with MoWs 2, 3, 5 and 7 return acidic phagolysosomes percentages significantly higher than those observed with *Candida* pretreated with PBS. Interestingly, this phenomenon is to be ascribed to the inhibition of fungal dimorphic transition. Indeed, fungi treated with such MoWs remain in a yeast form and they are rapidly associated with acidic phagolysosomes It is worth noting that the MoWs capable to impair *Candida* dimorphic transition and, in turn, intracellular compartment acidification contain either CHX or essential oils in their formulation. These results are in line with the literature reporting that alcohol- and/or CHX-containing MoWs have the highest antibacterial effects *in vitro* [43,44] and in the oral cavity [45]. Initial studies employing *Candida spp*. [46] have shown that CHX-containing MoWs are also effective against fungal cells, both in planktonic [47] and sessile forms [16,48]. Furthermore, in agreement with our present data, recent reports have shown that also MoWs containing essential oils have antifungal effects on several species of *Candida* [49]. Differently, the pretreatment with MoWs 1 and 4 does not impair *Candida* dimorphic transition and the fungus maintains its capacity to form hyphae as well as control group, irrespective of its intracellular and extracellular localization. Consequently, once phagocytosed, *Candida* can avoid phagolysosome acidification and in turn intracellular killing, probably via disturbance of the vacuolar membranes by the growing hyphae. We favour the idea that the presence of an anti-discoloration system (ADS) in the formulation of MoW 1 (which nevertheless contains 0.20% CHX, as well as MoWs 2 and 3), may partially interfere with CHX-mediated anti-fungal effects. Interestingly, upon incubation with MoW 1, only *Candida* capacity to form biofilm [16] and its susceptibility to phagocytes remain unchanged, while other virulence traits, such as adhesion and elicitation of proinflammatory cytokines, are affected. Once again, the results of fungal cell viability are in line with these data.

On selected experiments, MoWs containing either CPC or Triclosan have been used. CPC-containing MoWs have been reported to be more effective on the planktonic, rather than the sessile form of *C*. *albicans* [48]. Here, we strengthen and expand such data, by showing that only CPC-containing MoW 8 indeed impairs adhesion of the yeast form of *C*. *albicans* to both biotic and abiotic surfaces.

As for the streptococci employed in the present study, we have chosen several species acting as early colonizers in multispecies microbial biofilm formation on dental surface [20]. All these species may play a role, albeit an indirect one, in the onset of several pathologies affecting the oral cavity, including caries. When using CHX-containing MoWs, a significant impairment in bacterial biofilm production has been observed for 4 out of 6 species investigated, i.e., *S*. *salivarius*, *S*. *vestibularis*, *S*. *parasanguinis* and *E*. *faecalis*. A slightly lower effect has been observed upon treatment with MoW 5. Based on the fact that CHX-containing MoWs (1, 2, 3 and 7) and, although to a lesser extent, the essential oils-containing MoW 5 impair biofilm formation by the so-called “pro-cariogenic” bacterial species [50–52], we speculate that such MoWs may indirectly provide protection against carious lesions onset. CPC, which is included in the formulation of several toothpastes and MoWs, is known to have anti-plaque and anti-gingivitis effects [53]. In our hands, CPC-containing MoW 8 happens to impair biofilm formation by *S*. *salivarius* even more effectively than CHX-containing MoWs. In contrast, Triclosan-containing MoW 9 does not seem to have any effect, in line with previous data reporting lack of antimicrobial effects and the induction of bacterial resistance mechanisms by Triclosan-containing cosmetic products [54]. Differently, the treatment of *S*. *sanguinis* and *S*. *mitis/oralis* isolates with the MoWs does not affect their biofilm formation. Noteworthy, *S*. *sanguinis* acts as a natural competitor of *S*. *mutans*, by establishing spatial and temporal antagonistic relationships with several mechanisms [55]. In addition, *S*. *sanguinis*, *S*. *mitis* and *S*. *oralis* produce small molecules with antibiotic-like activity, capable to inhibit the growth of other potentially pathogenic microorganisms, such as *C*. *albicans* and *Staphylococcus aureus* [20,56]. As a direct evidence of their beneficial role, *S*. *sanguinis*, *S*. *mitis* and *S*. *oralis* have been found to be predominant on oral mucosae of healthy subjects [57]. Taking together all these data, we may speculate that the MoWs here employed are effective in contrasting caries onset since not only they reduce the colonization of bacterial species that (albeit indirectly) have a potential pathogenic effect, but also they do not damage bacterial species which are competitors of the cariogenic *S*. *mutans*.

Finally, in order to assess if MoWs may impact on *Candida*-streptococci interplay, we have co-infected TR146 oral epithelial cells with untreated *C*. *albicans* and MoWs-treated *S*. *salivarius*. In particular, *S*. *salivarius* isolate number 12 has been chosen, being its biofilm formation the most impaired by MoWs. Our results show that once exposed to MoW 1 and MoW 5, *S*. *salivarius* partially inhibits *Candida* biofilm formation. In particular, early biofilm (24 hours from infection) but not mature *Candida* biofilm (48 hours from infection) is slightly affected, the latter irrespective of the MoW employed. By these data, we conclude that *S*. *salivarius* treated with MoWs 1 and 5 delays *Candida* biofilm formation, but it fails to stop it completely.

MoWs affect several virulence traits of the oral microbiota, acting on both fungal and bacterial cells. According to our past [16] and present results, CHX- and essential oils-containing MoWs exhibit the most powerful anti-*Candida* effects impairing biofilm formation, adhesion, elicitation of proinflammatory responses and enhancing intracellular killing via acidic phagolysosomes. Also, viridans streptococci capacity to form biofilm is deeply affected. Notwithstanding its effectiveness, notoriously CHX has several side-effects (staining of teeth, loss of taste, numb feeling [58]) that have to be considered when employing MoWs containing this molecule. On the other side, CHX-free MoWs, which include fluorine-containing molecules in their formulation, provide some help in the process of enamel remineralization (and consequently in caries prevention) [59], but they may not ensure a direct protection against pathogenic microorganisms. CPC-containing MoW seems a promising alternative to CHX-containing MoWs, even though more in-depth comparative studies are required to confirm these data. Finally, the lack of effect of Triclosan-containing MoW 9 does not warrant further studies. In addition, Triclosan has recently been banned from some cosmetic products by the FDA in the USA, because of the concerns on its side effects on human health [54].

In conclusion, because of the complexity of the oral environment and of the possible side effects of the active molecules of their formulations, special attention should be used when choosing MoWs for prevention and/or treatment of oral pathologies, also according to the etiological agent likely involved.

## Supporting information

S1 Table Details of the mouthwashes employed in the study. Commercial names and composition (with indication of the main components) are provided.
(PPTX)
