# Supplementary material for: In vitro effects of commercial mouthwashes on several virulence traits of Candida albicans, viridans streptococci and Enterococcus faecalis colonizing the oral cavity
Source: PLoS One. 2018 Nov 15;13(11):e0207262. doi: 10.1371/journal.pone.0207262 (PMC6237365; doi:10.1371/journal.pone.0207262)
Supplement: S1 Table — Commercial names and composition (with indication of the main components) are provided. (PPTX) [file pone.0207262.s001.pptx]

## Slide 1
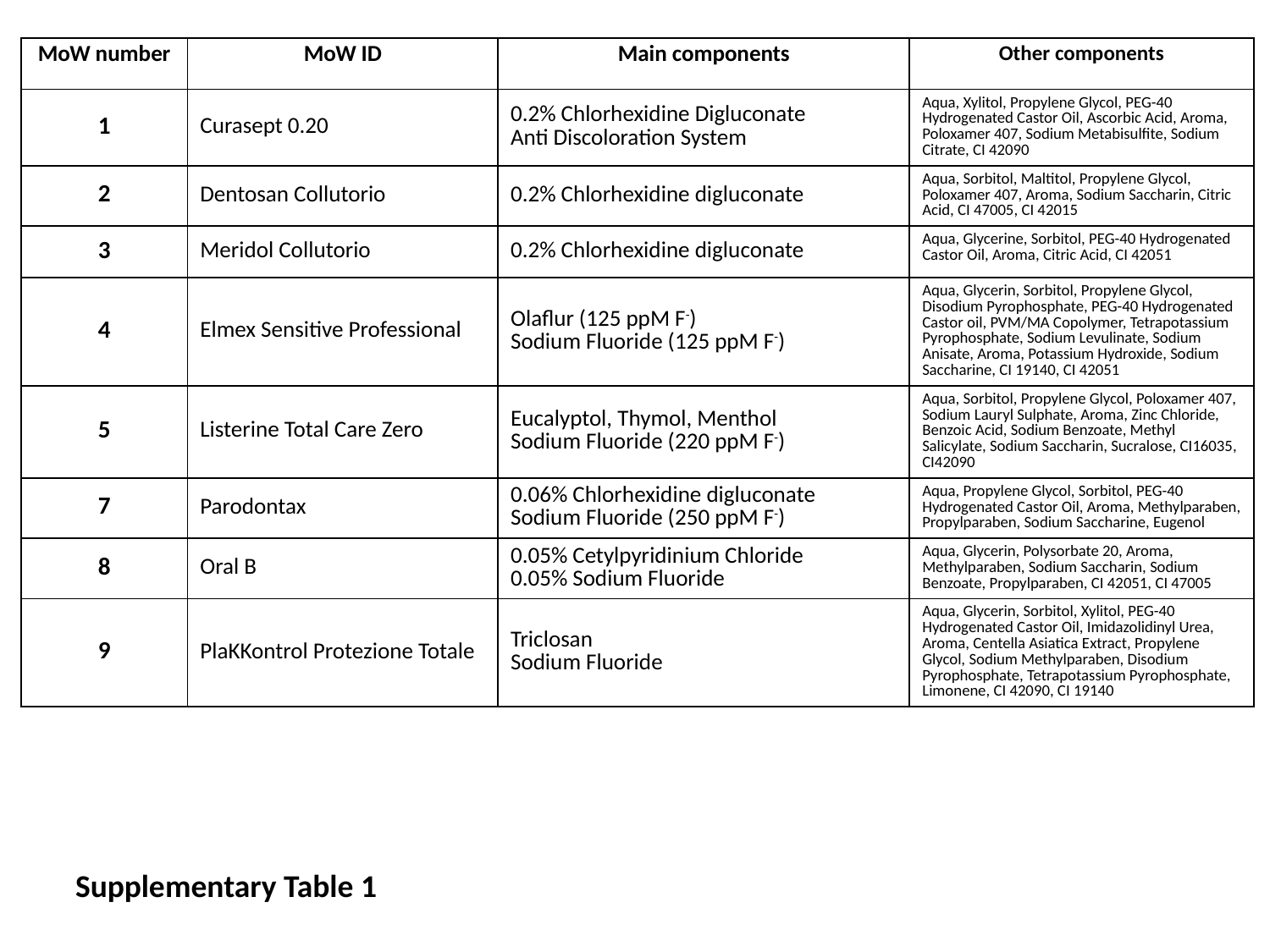

| MoW number | MoW ID | Main components | Other components |
| --- | --- | --- | --- |
| 1 | Curasept 0.20 | 0.2% Chlorhexidine Digluconate Anti Discoloration System | Aqua, Xylitol, Propylene Glycol, PEG-40 Hydrogenated Castor Oil, Ascorbic Acid, Aroma, Poloxamer 407, Sodium Metabisulfite, Sodium Citrate, CI 42090 |
| 2 | Dentosan Collutorio | 0.2% Chlorhexidine digluconate | Aqua, Sorbitol, Maltitol, Propylene Glycol, Poloxamer 407, Aroma, Sodium Saccharin, Citric Acid, CI 47005, CI 42015 |
| 3 | Meridol Collutorio | 0.2% Chlorhexidine digluconate | Aqua, Glycerine, Sorbitol, PEG-40 Hydrogenated Castor Oil, Aroma, Citric Acid, CI 42051 |
| 4 | Elmex Sensitive Professional | Olaflur (125 ppM F-) Sodium Fluoride (125 ppM F-) | Aqua, Glycerin, Sorbitol, Propylene Glycol, Disodium Pyrophosphate, PEG-40 Hydrogenated Castor oil, PVM/MA Copolymer, Tetrapotassium Pyrophosphate, Sodium Levulinate, Sodium Anisate, Aroma, Potassium Hydroxide, Sodium Saccharine, CI 19140, CI 42051 |
| 5 | Listerine Total Care Zero | Eucalyptol, Thymol, Menthol Sodium Fluoride (220 ppM F-) | Aqua, Sorbitol, Propylene Glycol, Poloxamer 407, Sodium Lauryl Sulphate, Aroma, Zinc Chloride, Benzoic Acid, Sodium Benzoate, Methyl Salicylate, Sodium Saccharin, Sucralose, CI16035, CI42090 |
| 7 | Parodontax | 0.06% Chlorhexidine digluconate Sodium Fluoride (250 ppM F-) | Aqua, Propylene Glycol, Sorbitol, PEG-40 Hydrogenated Castor Oil, Aroma, Methylparaben, Propylparaben, Sodium Saccharine, Eugenol |
| 8 | Oral B | 0.05% Cetylpyridinium Chloride 0.05% Sodium Fluoride | Aqua, Glycerin, Polysorbate 20, Aroma, Methylparaben, Sodium Saccharin, Sodium Benzoate, Propylparaben, CI 42051, CI 47005 |
| 9 | PlaKKontrol Protezione Totale | Triclosan Sodium Fluoride | Aqua, Glycerin, Sorbitol, Xylitol, PEG-40 Hydrogenated Castor Oil, Imidazolidinyl Urea, Aroma, Centella Asiatica Extract, Propylene Glycol, Sodium Methylparaben, Disodium Pyrophosphate, Tetrapotassium Pyrophosphate, Limonene, CI 42090, CI 19140 |
Supplementary Table 1
